# Does a truncated form of the transcription factor Ets1 exist in breast cancer cells?

**DOI:** 10.1038/sj.bjc.6602903

**Published:** 2005-12-06

**Authors:** J Dittmer

**Affiliations:** 1 Research Laboratory, Clinic for Gynecology, University of Halle, Ernst-Grube-Str. 40, 06120 Halle, Germany

**Sir**,

I have read with great interest the publication by Buggy *et al* (2004) on overexpression of the Ets-1 transcription factor in human breast cancer. Ets1, the founding member of the Ets family of transcription factors, is typically expressed in more advanced epithelial tumours and believed to play an important role in invasion (Dittmer, 2003; Hsu *et al*, 2004). Underscoring the importance of Ets1 in tumorigenesis, Ets1 has been shown to be an independent prognostic factor for breast cancer (Span *et al*, 2002).

Consistent with Ets1's importance for invasion, Buggy *et al* (2004) demonstrate that expression of Ets1 correlates with that of the protease uPA, a key enzyme for ECM degradation. Not only was the correlation found for p54 full-length Ets1 but also for a smaller 33 kDa protein that reacted with the anti-Ets1 antibody. The antibody, anti-Ets1 C-20 (Santa Cruz), that was used for these analyses recognizes the very C-terminus of the Ets1 protein and is a standard reagent for the detection of Ets1 in Western blot and immunoprecipitation assays (Vetter *et al*, 2005). By using the same antibody for the analysis of primary breast extracts, we also detected a 33 kDa protein in addition to the Ets1 protein (Figure 1A). The same protein was also present in extracts of breast cancer cell lines, such as MDA-MB-231 cells.

An Ets1 protein of this size could either be the not yet detected product of the doubly-spliced Ets1 RNA (Jorcyk *et al*, 1991) or an Ets1 degradation product containing the C-terminal part of the Ets1 protein. To distinguish between the two possibilities, we used the Ets1-specific antibody N-276 (Santa Cruz), which recognizes amino acids 55–70 of the Ets1 protein. This antibody should be able to interact with the theoretical double-spliced form, but not with an N-terminal truncated form of Ets1. It turned out that the N-276 antibody failed to interact with p33 indicating that p33 is not the doubly spliced form of Ets1 (data not shown). Hence, if p33 is indeed an Ets1 protein, it is likely to be an N-terminal truncated form of Ets1. Based on its size, p33 should harbour the DNA-binding domain, the regulatory exon VII domain, but only part of the activation domain of Ets1. Interestingly, Ets DNA-binding domains without functional activation domains act as trans-dominant negative proteins (Foos and Hauser, 2004). Hence, there is the exciting possibility that p33 is a naturally occurring trans-dominant negative form of the Ets1 protein that could have important regulatory function on Ets1 dependent gene expression.

In order to characterize the p33 protein, we performed a number of experiments. First, we wanted to know how changes in the expression of Ets1 affect the level of p33. We suppressed Ets1 expression by treating MDA-MB-231 cells with either an Ets1-specific siRNA or by PKC inhibitor calphostin C (Lindemann *et al*, 2001; Vetter *et al*, 2005). Unexpectedly, none of these treatments had an influence on the p33 protein level (Figure 1A). The C-20 anti-Ets1 antibody can be used to pull down native Ets1 protein in an immunoprecipitation experiment (Vetter *et al*, 2005). To analyse the ability of this antibody to interact with native p33, we labelled proteins in MDA-MB-231 cells with S-35 and separated the proteins on SDS-PAGE. While the full-length Ets1 protein could be visualized by this method, no p33 band could be detected (Figure 1A). We next analysed whether full-length Ets1 and p33 colocalize in the cell. Nuclear and cytosolic protein extracts were prepared from MDA-MB-231 cells and subjected to Western blot analyses. As expected, full-length Ets1 was primarily found in the nuclear extract (Figure 1A). In contrast, p33 is predominantly expressed in the cytosol. Next, we enriched the full-length Ets1 and p33 proteins by ionic exchange chromatography and subjected the proteins to partial tryptic digestion to determine whether similar protein fragments would be generated. The exon VII domain contains three hot spots for trypsin proteolysis leading to 23, 19 and 15 kD C-terminal fragments (Jonsen *et al*, 1996). Two of these fragments, the 15 and a 19 kDa peptides, could be visualized when our full-length Ets1 preparation was treated with trypsin (Figure 1B). However, none of these fragments were found when the p33 protein was digested with trypsin. We finally subjected the p33 protein to MALDI-TOF analysis. By using this method, the protein was determined to be annexin V. To confirm this result, we preformed a Western blot analysis with purified annexin V. Not only did the anti-Ets1 C-20 antibody interact with annexin V, but annexin V had the same apparent molecular weight in SDS-PAGE as the p33 protein (Figure 1C). These data indicate that p33 is not a truncated form of Ets1, but annexin V.

As found for p33, annexin V is primarily localized in the cytoplasm, but is also present in the nucleus (Sun *et al*, 1992). Annexin V is often used for detection of apoptosis as it binds with high affinity to phosphatidylserine which flip–flops to the outer leaflet of the plasma membrane in the event of apoptosis. Thanks to its interaction with phosphatidylserine, annexin V is able to inhibit the activity of membrane-bound PKC*α* (Dubois *et al*, 1998). PKC*α*, on the other hand, is a major regulator of Ets1 (Vetter *et al*, 2005). In this way, Ets1 and annexin V may even be linked. The observation by Buggy *et al* (2004) that the expression of their p33 correlates with that of uPA may suggest that annexin V and uPA are coordinately expressed in breast cancer cells.

## Figures and Tables

**Figure 1:**
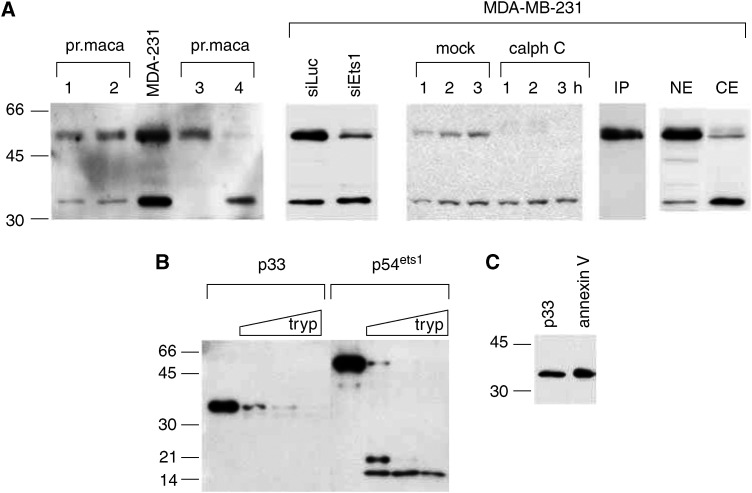
The p33 anti-Ets1 reacting protein is annexin V. (**A**–**C**) Western blot analyses with the anti-Ets1 (C-20) antibody. (**A**) Analyses performed on protein extracts from primary breast cancer biopsies (pr. maca) and MDA-MB-231 breast cancer cells. In an siRNA experiment, MDA-MB-231 cells were either transfected with an Ets1-specific siRNA (siEts1) or a control siRNA (siLuc). In a different set of experiments, MDA-MB-231 cells were either treated with calphostin C (calph C) or mock-treated. IP indicates an immunoprecipitation assay where S-35 labelled MDA-MB-231 protein extracts were incubated with an anti-Ets1 (C20) antibody-agarose conjugate to specifically precipitate Ets1 proteins which were then run on a SDS protein gel and visualized by exposure of the gel to an X-ray film. NE and CE designate nuclear and cytosolic extracts from MDA-MB-231 cells. (**B**) Chromatographically enriched p33 and Ets1 were partially digested with trypsin and resulting fragments determined by Western blot analyses by using the anti-Ets1 (C-20) antibody. (**C**) Chromatographically enriched p33 and purified annexin V were analysed by Western blot analysis by using the same antibody.
